# The undetected loss of aged carbon from boreal mineral soils

**DOI:** 10.1038/s41598-021-85506-w

**Published:** 2021-03-18

**Authors:** Geert Hensgens, Hjalmar Laudon, Mark S. Johnson, Martin Berggren

**Affiliations:** 1 Department of Physical Geography and Ecosystem Science, Lund University, Lund, Sweden; 2 Department of Forest Ecology and Management, Swedish University of Agricultural Sciences, Umeå, Sweden; 3 Institute for Resources, Environment and Sustainability, University of British Columbia, Vancouver, Canada; 4 Department of Earth Science, Vrije Universiteit Amsterdam, Amsterdam, The Netherlands

**Keywords:** Biogeochemistry, Carbon cycle, Biogeochemistry, Environmental sciences, Limnology

## Abstract

The boreal forest is among the largest terrestrial biomes on earth, storing more carbon (C) than the atmosphere. Due to rapid climatic warming and enhanced human development, the boreal region may have begun transitioning from a net C sink to a net source. This raises serious concern that old biogenic soil C can be re-introduced into the modern C cycle in near future. Combining bio-decay experiments, mixing models and the Keeling plot method, we discovered a distinct old pre-bomb organic carbon fraction with high biodegradation rate. In total, 34 ± 12% of water-extractable organic carbon (WEOC) in podzols, one of the dominating boreal soil types, consisted of aged (~ 1000 year) labile C. The omission of this aged (i.e., Δ^14^C depleted) WEOC fraction in earlier studies is due to the co-occurrence with Δ^14^C enriched modern C formed following 1950s nuclear bomb testing masking its existence. High lability of aged soil WEOC and masking effects of modern Δ^14^C enriched C suggests that the risk for mobilization and re-introduction of this ancient C pool into the modern C cycle has gone undetected. Our findings have important implications for earth systems models in terms of climate-carbon feedbacks and the future C balance of the boreal forest.

## Introduction

Since the last glacial maximum, the boreal region has acted as a net carbon (C) sink largely as a result of the northward expansion of forest ecosystems^1,2–4^. However, as the forest landscape ages, the strength of the sink decreases and the C exchange can reach equilibrium^5,6^. In recent decades the boreal region has seen the fastest increase in temperatures of all global forested areas, and this trend is expected to continue^7^. This has led to increased forest fires^8^, permafrost thaw^9,10^ and soil C loss^11^. Climate models suggest that the boreal C stock may decrease under future scenarios^12,13^. Increased anthropogenic disturbances as a result of an expected northward-moving agricultural limit^14^ or through intensified forest management^7^ could result in the increased mobilization of aged C to rivers^15^. Once released, aged terrestrial C can readily be decomposed in aquatic ecosystems^16–18^, leading to increased mineralization rates of organic aged C which can be seen as equivalent to the burning of fossil fuels^19^.

Approximately 95% of the boreal C stock is stored below ground as soil organic carbon (SOC)^20,21,22^. Peatlands and organic permafrost soils represent the most SOC dense areas^23^, but podzol soils have the largest spatial extent^24^ and thus hold a substantial and important part of the total boreal C stock. As such, the future C dynamics of the boreal forest are largely dependent on the fate of its soil C stock^25^. Until recently, C preservation in podzol soils was thought to be dependent on selective preservation of persistent organic matter^26^. However, recent advances in soil science have shown that limited microbial access to organic matter due to soil-matrix shielding of C determines long-term storage potential^27–30^. Thus, the idea that aged C in soils only consists of intrinsically recalcitrant compounds has been challenged^29,31–34^. Corroborating evidence for this include the finding that riverine aged terrestrial C is actively and preferentially utilized within the aquatic foodweb^16,17^. A better understanding of the losses and subsequent tracing of aged biogenic soil C is thus needed for predicting the future of the boreal C stock and the export of C across the terrestrial—aquatic interface.

**Figure 1:**
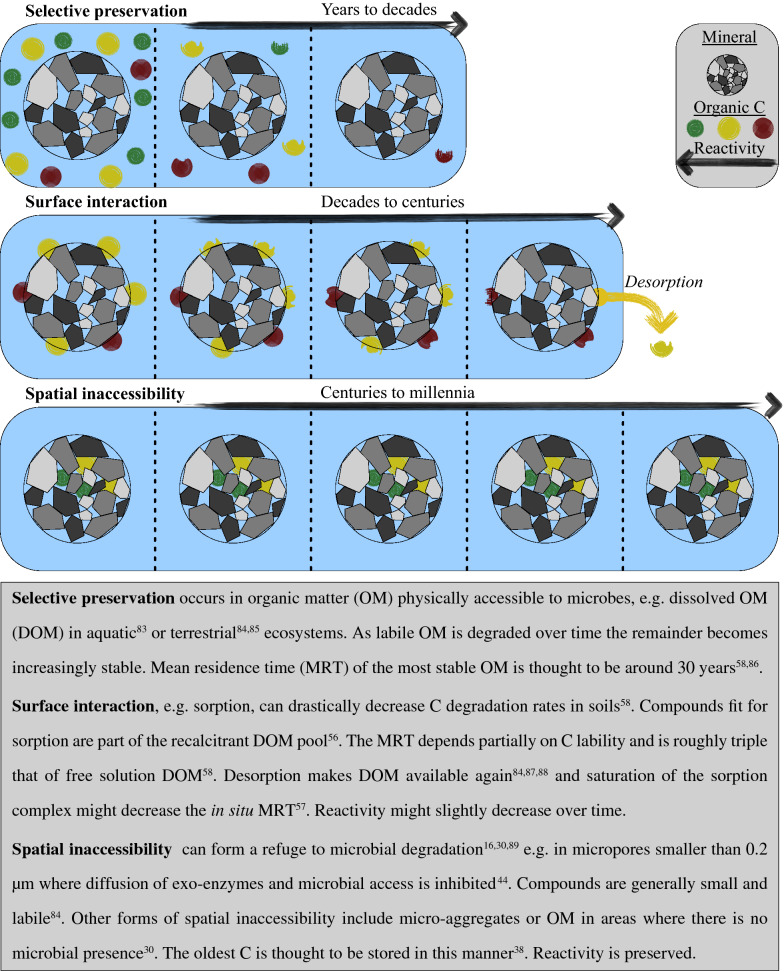
A conceptual overview of three different C preservation processes in mineral soils.

We here determined the radiocarbon age of SOC and the water-extractable OC (WEOC) of a boreal podzol profile in order to study the potential loss of aged C through soil C leaching. Although aged DOC exports have frequently been reported^15,35–40^, radiocarbon dating of mixed origin samples often is problematic because of the presence of modern C masking the old C signal. Following the nuclear bomb testing in the 50 s and 60 s, the standardized atmospheric Δ^14^C ratio in the northern hemisphere increased from − 20 to > 900‰ in a matter of years^41^. Average Δ^14^C signatures of mixed samples are thus unequivocally biased towards C fixed during modern post-nuclear times. This can mask the presence of aged C resulting in the false interpretation that terrestrial DOC primarily is of modern origin^42,43^. Because long term preserved aged C is less easily leached compared with modern C^44^, we hypothesized that leachates are biased toward elevated Δ^14^C signatures that mask old C fractions in the bulk WEOC. Aged C preserved through spatial inaccessibility, as recent views suggest, is expected to be more reactive once released than modern C preserved selectively (Fig. 1). Therefore, we performed an incubation study using the Keeling plot technique^45,46^ to separate the Δ^14^C signal of the labile aged DOC fraction from that of the bulk signal. A dark incubation was set up in triplicates for leachates from each soil horizon at 20 °C. Measurements of DOC and its Δ^14^C were done at fixed intervals after 1, 3, 6, 12 and 25 weeks. This approach was used to determine the isotopic C signature of respired C from the intercept of the relationship between Δ^14^C and the inverse C concentration at different time points of a DOC decay experiment. This allowed us to determine if the aged C was present in the labile pool, even though it could not be readily detected in the bulk signal. Using a mixing model approach, we then calculated the percentage of labile aged C present in the WEOC.

### Depleted Δ^14^C in soils and labile DOC

**Figure 2:**
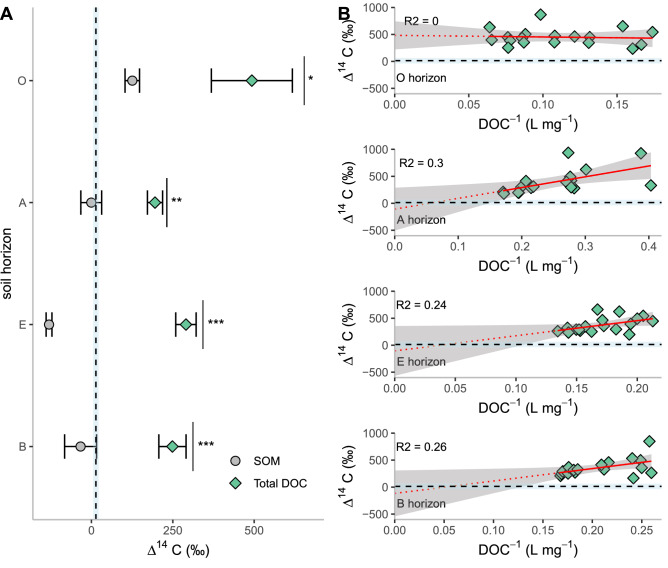
(**A**) The Δ^14^C signature of soil organic matter (SOM) and WEOC at time point 0, significance is given by *p < 0.05, **p < 0.005 and ***p < 0.001. (**B**) Keeling plots depicting the Δ^14^C signature as function of the inverse DOC concentrations. Note the y-axis intercepts, which represent the isotopic signatures of the degraded DOC. The straight dashed line represents the most current (2015) atmospheric Δ^14^C value of CO_2_. The 95% interval is given in grey.

**Figure 3:**
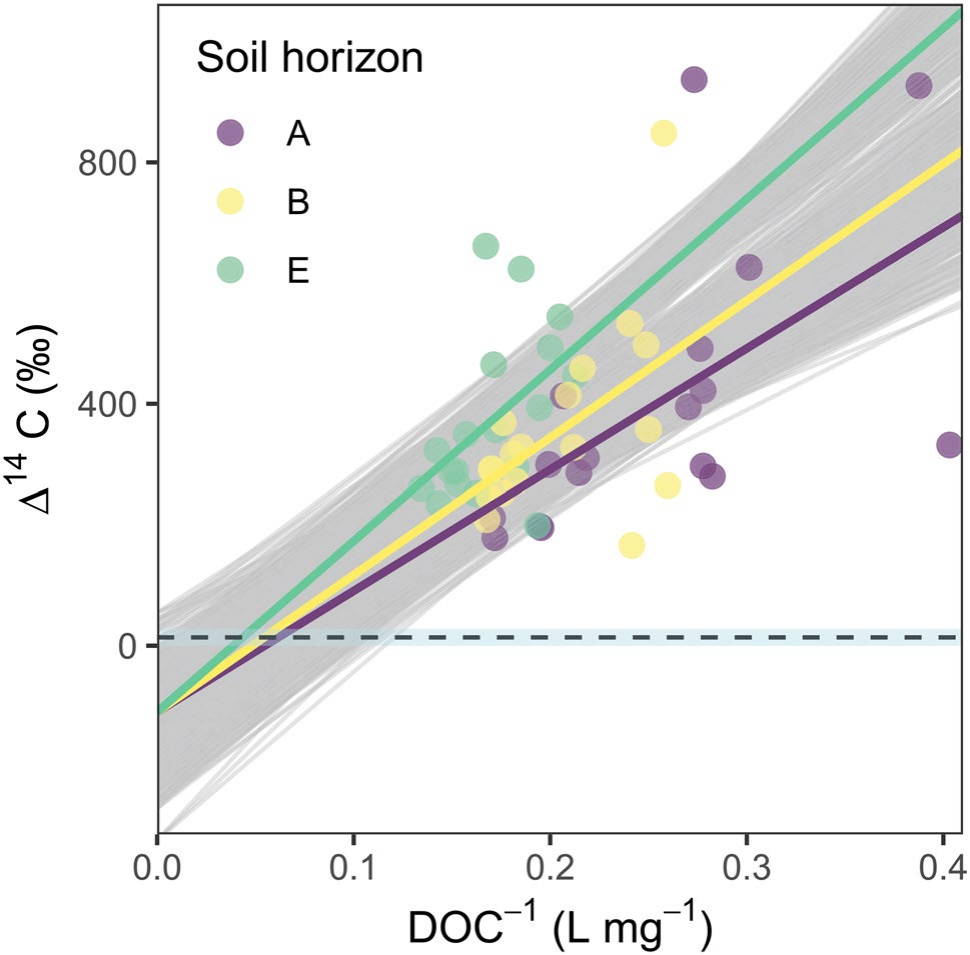
Results from the parametric bootstrapping of the Keeling plot intercepts, showing the first 200 of the 100.000 simulations (grey lines), the original data of the triplicate incubations (dots) and the original fitted multiple linear regression lines (colored lines corresponding to soil horizons). The horizontal black dashed line represents the most current (2015) atmospheric Δ^14^C value of CO_2_.

The Δ^14^C values of SOC were found to decrease with depth, ranging from modern (126 ± 22‰) in the O horizon to pre-nuclear in the A (− 0.498 ± 32‰), E (− 130 ± 9.0‰) and B (− 33.4 ± 49‰) horizon (Fig. 2A). The WEOC Δ^14^C signatures at time point 0 were elevated relative to the SOC values (O: *p* < 0.05, A: *p* < 0.001, E: *p* < 0.001, B: *p* < 0.01) showing a modern origin for WEOC in the O (493 ± 124‰), A (195 ± 23‰), E (290 ± 31‰) and B (249 ± 42‰) horizons (Fig. 2A). All WEOC values were higher than the most recently available atmospheric minimum Δ^14^C values (13.8‰ as reported by Graven and others 2017). Incubation led to a decrease in DOC over time (Supp. Fig. 1) as well as progressively increasing Δ^14^C values in all but the O horizon DOC extracts (Fig. 2B). Using the Keeling plot method, we showed depleted pre-nuclear Δ^14^C signals for the labile portion of DOC in the A (− 108 ± 186‰), E (− 105 ± 217‰) and B (− 116 ± 200‰) sub-horizons, and modern values for the O (482 ± 123‰) horizon (Fig. 2B). We further constrained the sub-soil intercept estimate using parametric bootstrapping on a multiple linear Keeling regression (R^2^ = 0.31) with only an interaction effect for soil layer. The average constrained Δ^14^C signal of the intercept was − 109‰ (± 71.6) with 95% of the simulated intercept values < 12.7‰ and 90% < -14.5‰ (Fig. 3). Using a mixing model (Eq. 1) with the same bootstrapping approach, we calculated that on average 34.2% (± 12.2) of the extracted WEOC consists of aged C for all mineral soil horizons (A: 43.1 ± 6.5%, E: 18.6 ± 3.1%, B: 40.9 ± 5.4%).

**Figure 4:**
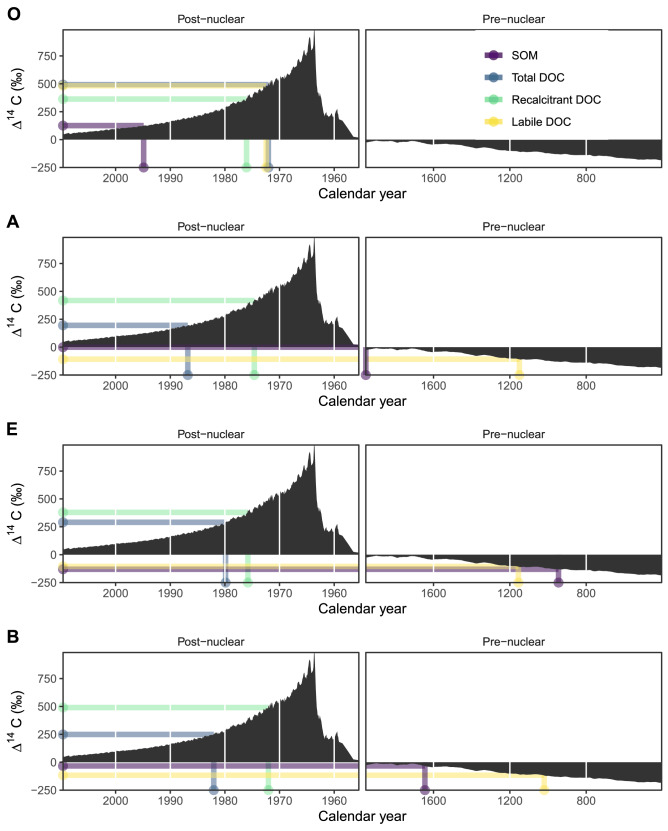
Observed average Δ^14^C projected on a timeline of historical atmospheric values for different soil organic matter (SOM) and dissolved organic carbon (DOC) fractions; SOM (purple), total (blue), recalcitrant (green) and labile (yellow) DOC.

Projected on historical atmospheric Δ^14^C values of the northern hemisphere^41^, the average radiocarbon age of the SOC correspond to atmospheric Δ^14^C concentrations of 1994 (O), 1954 (A), 945 (E) and 1645 (B) (Fig. 4). The WEOC Δ^14^C values relate to roughly the 1980s (A, E and B) or 1972 (O). The Δ^14^C signal of the resistant DOC remaining after the incubation had an average radiocarbon age originating in the 1970s. The labile DOC, on the other hand, showed Δ^14^C values indicating an average origin before year 1150 for sub-soil layers and an average origin in the 1970s for the O horizon.

### Discriminatory leaching of post nuclear C hides aged portion

Despite the SOM Δ^14^C signals suggesting old soil C in sub-soil layers, the extracted DOC had elevated Δ^14^C signals (Fig. 2) due to discriminatory leaching favouring the extraction of modern C. This is in line with previous findings of elevated Δ^14^C signals in DOC as compared to SOM^47^ and in particulate organic carbon (POC)^36^. Aged soil C has previously been linked to small molecules^31,38^, microbial material^48–50^ or small to large scale aggregates of C^32,51^ tightly interconnected within the soil matrix. Thus, the older soil C fraction has a greater resistance to leaching in comparison to the modern C fraction, which might originate from desorption or free soil water DOC.

Figure 2A shows that WEOC has a higher ^14^C content than SOC in all horizons. For the O (humus) horizon this implies that WEOC is older than SOM as the ^14^C values are post-nuclear whereas for the subsoil horizons WEOC is younger than SOM as the ^14^C values of SOM are pre-nuclear. This means that older C is preferentially leached in the O horizon. We found that the amount of DOC leached per gram of soil was about twice as high for the A horizon as the O horizon (data not shown). This indicates that successive decay of the humus (i.e. O) layer as it grades into the A layer leads to increased leaching. This is in accordance with studies showing that leaching from pine litter, the main component of the O horizon in this study, increases as decomposition advances^52,53^. The Δ^14^C SOC signal of the O horizon indicates an average origin of roughly 20 years ago. While this is relatively old for humus, the age is within the range (16: > 45) and the Δ^14^C signal is lower than previously reported in central Sweden^54^.

### Preferential decay of aged DOC

The incubation study showed a preferential decay of aged C in the extracted DOC. Although the age of the labile DOC has relatively high uncertainty, there is a less than 5% likelihood that the labile DOC was fixed under recent atmospheric Δ^14^C values^55^. In fact, only during a short period in the early 1950s do atmospheric Δ^14^C values (− 22.3 to 25.2‰)^41^ overlap with the 95% interval of the intercept (Fig. 3). This can be compared to thousands of years of fixed C with Δ^14^C signatures that fit > 90% of the simulated intercepts. Thus, the difference in the labile and recalcitrant Δ^14^C clearly places the time of fixation for the labile and the recalcitrant DOC prior to and during the bomb peak, respectively. However, it should be pointed out that both the labile and recalcitrant DOC are mixtures of C of different ages resulting in average Δ^14^C signatures that can be distinguished and interpreted in terms of an average age. As C is produced and stored continuously in soils it is likely that an unknown amount of modern C also was present in the labile WEOC. The mixing of these sources can explain the large variability seen in Δ^14^C during the incubation and the large uncertainty in the intercepts. Simultaneously, this implies that the aged C is potentially several thousands of years old, but only shows an average radiocarbon age of ~ 1000 years before present. These results are in accordance with a growing body of literature suggesting aged soil C consists, at least partially, of labile compounds once released^31,32,38^, as well as the observation that aged C exported to rivers is readily bioavailable^17,18^.

Selective preservation and surface interactions are known to result in soil retention of recalcitrant DOC^56^, but this retention is not necessarily long term^57^. In this regard, previous studies do not contradict our results that point to the presence of aged labile DOC in the water extractable fraction. Laboratory experiments suggest a mean residence time of 37–91 years^58^ for surface sorbed DOC, or shorter (4–30 years) if considering the saturation of the sorption complex^57^, which is well within the range of the radiocarbon ages we found for recalcitrant C (Fig. 4). Although some modelling studies suggest the presence of aged sorbed C in soils^59,60^, this might be due to definition issues which lack or blur the separation between sorption through surface interaction and e.g. aged detrital microbial material within aggregates or otherwise physically excluded C.

Nevertheless, direct sorption of C to mineral surfaces has been proposed to lead to long term C sequestration, possibly leading to recalcitrant fractions in aged soil C^57^. Although it should be noted that direct sorption to mineral particles is limited compared to the total sorptive capacity of soils^56,61,62^. As the direct C-mineral sorption consists of some of the strongest bonds^57,61^, it is unlikely that these compounds were extracted in our relatively mild extraction scheme^44,51,63^. It is also not likely that such compounds would be exported to rivers in significant quantity, evidenced by the finding that hydrophobic terrestrial DOC in rivers has a more modern origin compared to the non-hydrophobic part^38^. This means that within soils there might be a considerable aged DOC fraction that is inherently recalcitrant and/or is inactive because of its resistance to leaching.

### Export of labile aged C might go undetected

This study provides, to our knowledge, the first evidence and quantification for the masking of aged labile C leached from boreal mineral soils. Previous studies have provided evidence of old C exported from thawing permafrost, retreating glaciers and urbanized areas^35,40^. However, a wide range of studies have demonstrated the predominantly modern age of riverine C^16,64^, and a positive relationship between discharge and the Δ^14^C signal^39^. This has resulted in the view that little to no aged OC is exported from these systems under baseflow^16^ or during high discharge events^39^. Our results found that ~ 34% of the extracted soil DOC consisted of aged C with a mean age of ~ 1000 years, although this could never have been deduced from its bulk Δ^14^C signal. These findings extend previous evidence for nuclear Δ^14^C masking^42,43^, and are in line with the preferential decay of aged C found in various aquatic^16–18^ and terrestrial^32^ ecosystems, suggesting the export of aged C might be more common than generally thought.

While our study does not conclude whether or how much aged labile C is exported to rivers, there are a variety of processes known to disrupt aged C in soils^65^. Agriculture and urbanization can cause a breakup of soil structure resulting in immediate and sustained mobilization of aged soil C^15,32,36,38^. A change in vegetation^66^ or nutrient supply^67^, an increase in bioavailable carbon^68^ and the draining of waterlogged soil^65^ have all been linked to the rapid destabilization of aged soil C. In addition, the warming of boreal soil layers (as an effect of seasonal warming^69,70^, or after the removal of vegetation through natural fires^71^) has been shown to result in the release of pre-nuclear C. Many of these drivers are thought to become more severe as a result of climate change in the boreal region^7,8,72^. Although evidence against the climate-induced destabilization of aged soil C has been proposed^64,73^, possible nuclear masking in bulk Δ^14^C measurements of rivers cast doubt on validity of conclusions that the C is exclusively modern. Our results show that a modern bulk Δ^14^C signature is not sufficient to conclude that no aged C is being lost, meaning that soil export of aged C has likely gone undetected. This suggests that the mobilization of aged soil C to aquatic systems and subsequently the atmosphere, which has been observed locally in lakes^18^, is likely more widespread than previously thought and may contribute to the globally significant emissions of greenhouse gases from inland waters.

## Methods

### Field-site and sample handling

Soil cores were taken from podzol soils in a pine (*Pinus sylvestris*) dominated boreal forest in the Krycklan catchment (64° 14ʹ N, 19° 46ʹ E), northern Sweden^74^. The location is one of the most studied field site in the north and has been shown to be a well-represented location for the boreal biome^75^. The organic soil content ranges from ~ 5% in the A horizon to < 2% t lower depths^76^. The soil is a well-developed iron podzol on sandy till with a varying depth up to ~ 75 cm. The humus (O) layer is up to 5 cm thick on top of a sandy bleached E-horizon and a thick red-colored B-horizon^76^. The bedrock is a Sveco-fennian metasediments-metagraywacke^77^. The area has a cold temperate humid climate with persistent snow cover from November to April and a mean annual precipitation of 640 mm (1981–2010), 35% of which falls as snow^74^.

Soil cores were taken in August 2016 using a tubular soil sampler and split according to soil horizon. Samples were dried at 60 °C for at least 5 days and stored at − 4 °C before analysis. A minimum of 3 cores were combined into 1 composite sample, for a total of 4 composite samples representing the soil horizons. Small rocks and large roots were removed from subsoil samples. Composite samples were homogenized and divided across 3 replicates per soil horizon before further analysis (total number = 12). Three soil samples were sent for ^14^C analysis per horizon.

### WEOC extraction and incubation

We performed pure water extractions on replicates of soils with a varying soil:water ratio for each horizon in order to extract enough C for the subsequent DOC incubation experiment. Dried soils were added as 25, 55, 320 and 240 g per liter (O, A, E and B respectively) in 1 or split among 2 HDPE Nalgene bottles and shaken for 48 h at 140 round per minute lying down, but firmly attached on a platform shaker. No alkaline solution was used in order to preserve a natural leaching and following incubation solution and prevent bias through alkaline leaching^29^. Samples were vacuum filtered using 0.7 µm glass fibre filters overnight and stored at 4 °C for two days before the start of the incubation. Filtrates were diluted using purified water to 12.5 (O) and 7.5 mg C L^−1^ (A,E;B) in 6 L HDPE jerrycans for the incubation study. Jerrycans were stored at 20 °C in a constant room and shaken before sampling.

### Carbon and radiocarbon measurements

Before dilution, DOC was measured on a Shimadzu TOC V-CPN analyzer, using the Nonpurgeable Organic Carbon mode^78^. After dilution and during the incubation, DOC was measured using an OI analytical Aurora TOC analyzer (analytical precision: ± 0.2 mg C L^−1^). For every timepoint 400 ml samples were taken for Δ^14^C analysis, acidified to pH < 2 and frozen in 1 l round bottom glass flasks at − 24 °C. Samples were then freeze dried, re-dissolved in a 40 ml alkaline purified water solution and freeze dried again in clear 40 ml screw top vials. Vials used for freeze drying were covered with 0.7 µm glass fibre filters to prevent contamination.

Samples were sent to the Poznan Radiocarbon Laboratory for Δ^14^C analysis. Samples were pre-treated^79^, combusted and graphited^80^ and measured using a Compact Carbon AMS^81^.

### The Keeling plot method

The Keeling plot method^45,46^ was used to determine the isotopic signature of the labile fraction of the WEOC. In the method, the inverse of the decrease of DOC over time is plotted against the Δ^14^C of the WEOC and its related change over time^46^. The intercept of this regression gives the isotopic signature of the C that either disappears or is added. Originally, the method was used to determine the Δ^13^C signal of anthropogenic C emitted to the atmosphere^45^. Since then the method has been used to investigate the Δ^13^C signature of sources contributing to ecosystem respiration^46^, while a few studies have used it for Δ^14^C^17^. In this study the method was used to focus on the C that disappears.

### Statistical analysis and parametric bootstrapping

Statistical analysis were performed using the R core program^82^. A Welch’s two sample one-sided t-test was used to test the significance of the difference between the Δ^14^C signature of SOC and WEOC. Regressions for the Keeling plots were made using the *lm* function. Throughout the text uncertainty is given as one standard deviation, unless otherwise specified.

Parametric bootstrapping was done using the *bootstrap* R package. A multiple linear regression using a shared intercept and soil type as an interaction effect was fitted on simulated Δ^14^C and inverse DOC data. Normally distributed Δ^14^C data was simulated using the coefficients and residuals of the original linear regression, differentiating between subsoil type. The inverse DOC data was randomly simulated between the minimum and maximum inverse DOC data of the original dataset. A total of 100,000 simulations were performed. Similarly, a non-parametric bootstrapping was done to calculate the fraction of aged labile DOC (α) using Eq. (1) for each subsoil type:

1 $$\Delta^{14} C_{0} = \alpha \Delta^{14} C_{Intercept} + \left( {1 - \alpha } \right)\Delta^{14} C_{180}$$

where Δ^14^C_0_ and Δ^14^C_180_ are the average original data bulk Δ^14^C signals of WEOC at time 0 and after 180 days respectively and Δ^14^C_intercept_ is the simulated intercept Δ^14^C signal.

## Supplementary Information

Supplementary Figure

## Data Availability

The data that support the findings of this study are available from the corresponding author upon reasonable request.
