# Supplementary material for: The undetected loss of aged carbon from boreal mineral soils
Source: Sci Rep. 2021 Mar 18;11:6202. doi: 10.1038/s41598-021-85506-w (PMC7973788; doi:10.1038/s41598-021-85506-w)
Supplement: Supplementary file 1 — Supplementary Figure [file 41598_2021_85506_MOESM1_ESM.pdf]

The undetected loss of aged carbon from boreal mineral soils

Geert Hensgens[1*], Hjalmar Laudon[2], Mark S. Johnson[3], and Martin Berggren[1]

[1] – Department of Physical Geography and Ecosystem Science, Lund University, Lund Sweden

[2] – Department of Forest Ecology and Management, Swedish University of Agricultural Sciences, Umeå, Sweden

[3] – Institute for Resources, Environment and Sustainability, University of British Columbia, Vancouver Canada

[*] – Now at Department of Earth Science, Vrije Universiteit Amsterdam, Amsterdam the Netherlands

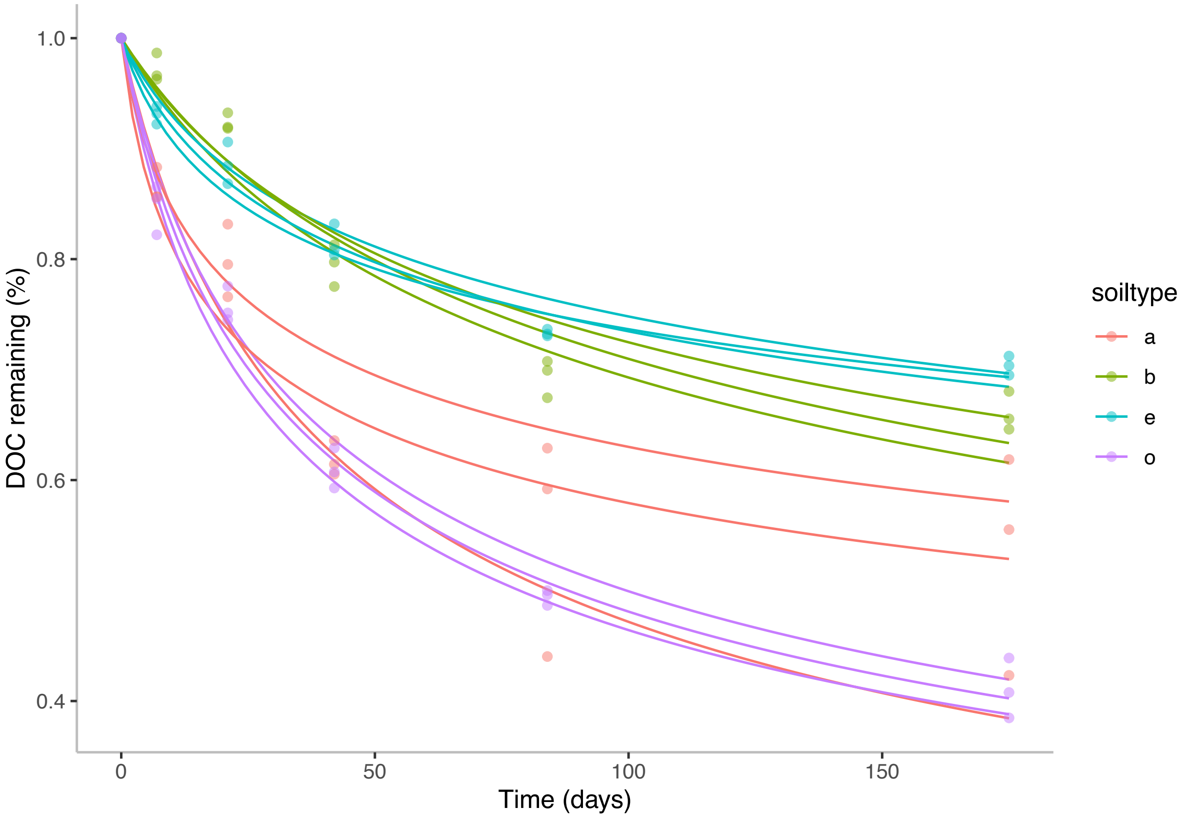

Supp. Fig. 1: DOC remaining (%) during the incubation of the WEOC from the soil horizons. Shown are the individual incubations with the lines representing the reactivity continuum model: $\frac{DOC_t}{DOC_0} = \left(\frac{a}{a+t}\right)^v$*, where a is a rate parameter and v is related to the shape of the distribution of decay rates at different times.*
